# isqg: A Binary Framework for *in Silico* Quantitative Genetics

**DOI:** 10.1534/g3.119.400373

**Published:** 2019-06-14

**Authors:** Fernando H. Toledo, Paulino Pérez-Rodríguez, José Crossa, Juan Burgueño

**Affiliations:** * International Maize and Wheat Improvement Center (CIMMYT), Km 45 Carretera México-Veracruz CP 56130, El Batán, Texcoco, Edo. de México, México and; † Colegio de Postgraduados CP 56230, Montecillos, Edo. de México, México

**Keywords:** Algorithms, Computational, Biology, Recombination, Simulation

## Abstract

The dna is the fundamental basis of genetic information, just as bits are for computers. Whenever computers are used to represent genetic data, the computational encoding must be efficient to allow the representation of processes driving the inheritance and variability. This is especially important across simulations in view of the increasing complexity and dimensions brought by genomics. This paper introduces a new binary representation of genetic information. Algorithms as bitwise operations that mimic the inheritance of a wide range of polymorphisms are also presented. Different kinds and mixtures of polymorphisms are discussed and exemplified. Proposed algorithms and data structures were implemented in C++ programming language and is available to end users in the R package “isqg” which is available at the R repository (cran). Supplementary data are available online.

The complexity and organization based on dna structure were evolutionarily optimized to faithfully store and transfer biological information (Bancroft *et al.* 2001). This complexity requires a proper computational representation in order to increase storage capacity, reduce the memory footprint and the run time of analyses. The fundamental unit for computer data are the binary digit (bit), just as the nucleotides are for dna. In this regard, each nucleotide may be represented as binary droplets *i.e.*, mapping {a, c, g, t} to {00,01,10,11}, respectively. Thus, the bitset 01101100 matches to the dna sequence cgta.

This analogy allows straightforward *in silico* representation of genetic polymorphisms by bits. On the other hand, a series of experiments have proved the value of synthetic dna as an alternative for storage. However, any dna-based storage system must own the capacity to retrieve the properties of the stored information (Erlich and Zielinski 2017). Computational representation of genetic data must also cater to depict the processes that drive the inheritance and genetic variability. This is especially important whenever computers are used for simulations.

Simulations are invaluable to the genetic research, in which computer programs are used to abstract complex models and the replications of pseudo data incorporate an inherent stochasticity common to genetic mechanisms (Hoban *et al.* 2012). Even when numeric or analytical approaches cannot be accessed, the simulations can be employed for inference, prediction and evaluation of new methods (Peng *et al.* 2015). To do so, an efficient abstraction of computational representation of the genetic variability is required.

Genetic information at sequence-level from various platforms is usually compressed by methods that are strongly linked to binary representation (Li *et al.* 2009). Methods for compressing genomic data into binary encoding are already being used by high-performance applications (Abecasis *et al.* 2002; Purcell *et al.* 2007). These representations, however, were designed with analytical purposes without taking account the dna structure, *i.e.*, the linkage phase between strands. It must be noted that simulations of meiosis, mutation and more recently, gene editing, are dependent on the strand base structure of the dna.

This paper introduces a new binary encoding of genome-level data that preserves the strand-based structure. Generalizations are provided to support a wide range of known polymorphisms. The algorithms to simulate the generation of variability are presented in terms of bitwise operations. The algorithms and data structures are available to users as an R package “isqg” which is available at the official R repository (cran).

## Methods

An oligonucleotide is a linear sequence of loci (*l*), each one indexed by a genome position (*x*), which may be associated with a physical or map coordinates. Thus, a binary representation of a dna strands consists of a set of bits ($l_{1},l_{2},\ldots ,l_{i}$) assigned to genome positions ($x_{l_{1}},x_{l_{2}},\ldots ,x_{l_{i}}$). Each bit has only two states, while a collection of *n* bits expands to $2^{n}$ states. In this sense, a locus represented by a single bit is biallelic and may be seen as single-nucleotide polymorphisms. Therefore, a collection of bits associated with the same position may represent different kinds of polymorphisms.

Homologous recombination over diploids is a universal mechanism in which sister chromatids pair with each other and undergo exchange of their contents. Considering two binary sets (*α* and *β*) as both dna strands, the mosaic recombination (*δ*) can be obtained by bitwise operations. To do this, another set (*γ*) must be introduced, which works as a bit mask. This mask controls the flow of the content between both chromatids. It works as a logical conditional statement such as an if-else block and thus, allow the composition of the mosaic between both chromatids *i.e.*, an 1 entry in *γ* means the bit is taken from *α*, while a 0 implies it is taken from *β*. As *α*, *β*, *δ* and *γ* are binary sets, a single bitwise statement must be performed combining the operators and (&), or (|) and not (∼), that is:

δ←(γ&α)|(∼γ&β). (1)

In the simplest case, with *k* biallelic loci, all $2^{k}$ masks are equally probable, *i.e.*, the content in *δ* is independently chosen between *α* and *β* with equal probabilities. To contemplate the linkage between loci close to each other, restrictions on the probabilities of the masks, conditionally on the coordinates, must be assigned *e.g.*, through recombination models (Copennhaver *et al.* 2002, see references therein). Regardless the recombination model, given the positions where the events occur, binary operations allow building the corresponding mask. For each event, the locus with the smaller position, greater than or equal to the chiasma position, is found (upper bound). The index of this locus is used to right shift (≫) a replete binary set, resulting in the bit mask for that event. A recursive chain of exclusive or (xor/∧) assignments over those masks build the mask merging all events:

**Algorithm 1**: Build bit masks for homologous recombination

1: **procedure** RecombinationMask(*chiasmata*, *map*)

2: *mask* ←zeros ;

3: **if** size(chiasmata)>0** then**

4: breaks←0 ;

5: **for** *chiasma* **in** *chiasmata* **do**

6: breaks←upperBound(chiasma in map) ;

7: event←ones≫breaks ;

8: mask←mask ∧ event ;

9: **if** $\mathrm{getRandom}{\left(\right)}_{\left[0;1\right]}>0.5$** then**

10: mask← ∼mask ;

11: return mask ;

The independent assortment of chromosomes also contributes to the generation of variability. Equation 1 considers all loci belonging to the same linkage group. Therefore, when performing the segregation in each chromosome, the independence among them is ensured through a random process that flips the mask (*γ*) beforehand, *i.e.*, ones turn zeros and vice-versa, which is carried out by the not operator (∼) (Lines 9-10 of Algorithm 1).

**Figure 1:**
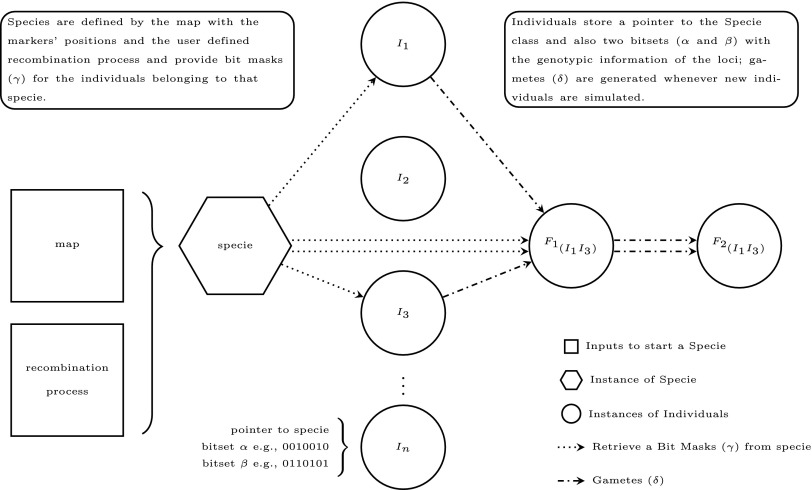
Schematic representation of the classes’ hierarchy implemented in “isqg”.

The construction of bit masks is independent of any genetic content, using only the positions of the loci and chiasmata as inputs. This feature allows the individual to be stored only by its dna strands. As gametes are requested, the masks are retrieved from the generator, common to all individuals. In this sense, any recombination models can be employed, and users are able to define their own model to support *e.g.*, recombination hotspots (Figure 1).

Species are often defined as a group of organisms in which any pair of individuals can produce fertile offspring. It can also define specie by identifying common dna features such as the karyotype. In this sense, it is defined as “specie” the generator class of objects which holds the needed information to link different individuals that share the characteristics at the dna level and can produce fertile offspring (Figure 1). The name given for this abstraction adhere to the biological definition and makes a straightforward relation between real and *in silico* information.

In addition, as recombination depends only to the loci’s positions, the linkage among a collection of bits assigned to the same position will never break, working as a single loci. This framework also supports a mixture of different types of polymorphism coexisting together, such as the one at the sequence-level or those with multiple alleles, which may unveil other understanding about the association of snps (biallelics) with complex quantitative traits driven by multi-allelic factors.

Mutations and/or gene editing changing the state of locus *i* from parent to offspring can also be performed by binary operations. Given the index of the loci to be changed (edited or mutated), a right shift on an empty bitset results in a bit mask for this event. Then, an exclusive or (xor/∧) operation between the mask and a DNA strand will toggle the bits where the event occurs. This procedure can be implemented analogously to Algorithm 1.

### Data Availability

Software are released under a public license (gpl-2); source codes and binary version of the software are available at the official public repository of R packages (cran): https://cran.r-project.org/package=isqg. The authors affirm that all data necessary for confirming the conclusions of the article are present within the article, figures, and tables. Supplemental material available at FigShare: https://doi.org/10.25387/g3.8162051.

## Results

A detailed description of “isqg” can be found in its documentation. Below we present two case studies to highlight the “isqg” features. First, it is presented how users can define their own recombination process to be used over simulations. After, a more realistic quantitative genetic simulation is performed.

### Custom Recombination

Example 1 show a situation where after the package is loaded, the example data (ToyMap) is loaded and an specie is started. Then, two completely contrasting individuals are initialized and the $F_{1}$, $F_{2}$ as well as recombinant inbred lines (double-haploids) are generated from the $F_{1}$. Genotypic data from the simulated individuals are then retrieved as numeric or keeping the phase information.

**Example 1:** Simple simulation of crosses and double-haploids with “isqg”

> library(isqg)

> data(ToyMap)

> *## start specie given the map*

> spp <- set_specie(ToyMap)

> *## start some ”founders” individuals from specie*

> AA <- spp$founder(code = ”AA”)

> aa <- spp$founder(code = ”aa”)

> *## making some crosses (cross*, *selfcross and dh)*

> *## are accessible by standalone function and R6 methods*

> F1 <-cross(n = 1, AA, aa) # the hybrid

> F2 <- F1$selfcross(n = 1E3) # segregating population

> RILs <- F1$dh(n = 1E3) # inbreeds

> *## retrieving the genotypic data*

> M <- genotype(RILs) # as numeric

> N <- F1$genotype(phase = TRUE) # as character (phased)

By default, instances of the species are started giving as input the map with the genomic positions in Morgans (*M*) and the meiosis recombination is performed through a count location process (Karlin and Liberman 1978). However, other recombination models can be employed thus, users can extend “isqg” capabilities. To do so, when initializing species as in Example 1, a function implementing the recombination process must be provided. As an example the authors share as a supplemental material a couple of commented C++ implementations for recombination processes in which markers segregate independently regardless of their position in the map as well as where recombinations happen in hotspots.

Having the extension code as a file *e.g.*, “Independent.cpp”, it can be seamlessly compiled and linked to R through the Rcpp package (Eddelbuettel and François 2011). Thus, users can simulated data according to any recombination model. Example 2 shows how users can check the behavior of the custom independent segregation in comparison with the standard count location process.

**Example 2:** Including and using extension for custom user-defined recombination process

> library(isqg)

> data(ToyMap)

> *## standard specie as in Example 1*

> standard <- set_specie(ToyMap)

> *## sample 100 gametes from standard process*

> standard$gamete(100)

> *## require Rcpp package*

> library(Rcpp)

> *## compile and link Example 2 at file Independent.cpp*

> sourceCpp(file = ”Independent.cpp”, rebuild = TRUE)

> *## specie with custom recombination*

> custom <- set_specie(ToyMap, meiosis = indepp())

> *## sample 100 gametes from custom process*

> custom$gamete(100)

### Quantitative Genetics Simulation

Considering a breeding scenario where $F_{2:3}$ progenies are generated from two contrasting parents. One question that may arise is how different are the predictions of the breeding values when it is used the realized genomic kinship (matrix G), taking account the Mendelian samplig, or the resemblance between individuals (matrix A) *i.e.*, the coefficient of identity by descent. To do so, a specie is initialized with a genetic map having fives chromosomes each one with 1000 equally spaced biallelic loci and a quantitative trait is defined with zero mean and additive effect of one for all loci. From this settings, two contrasting individuals and the $F_{1}$ are also initialized (Example 3).

**Example 3:** Initializing structures and data for a simulation with “isqg”

> *## generating map: 5 chromosomes and 1000 ”genes” per chromosome*

> map <- expand.grid(chr = 1:5, pos = seq(0, 2, length.out = 1000))

> map$snp <- paste0(”s”, 1:(5 * 1000))

> *## initializing ”specie” and ”infinitesimal” quantitative trait*

> spp <- set_specie(map)

> trait <- set_infty(spp, m = 0, a = 1, d = 0)

> *## initializing two contrasting parents and the F1*

> P1 <- spp$founder(code = ”AA”)

> P2 <- P$mirror()

> F1 <- cross(n = 1, P1, P2)

After that, one may define an R function to simulated the above mentioned breeding scenario:

- generating inbreeding generations *i.e.*, $F_{2}$ population with *N* individuals as well as $F_{2:3}$ progenies with *P* individuals each;
- getting the true breeding values regarding the defined quantitative trait and simulating phenotypes adding random deviates accoring to the a desired heritability ($h^{2}$);
- genotyping the individuals and obtaining the realized kinship matrix;
- fitting the mixed models with both kinds of information (realized kinship and identity by descent); and
- collecting and delivering the estimated additive and residual variances.

Replications of the simulated scenario are then obtained in which the collected statistics can be further analyzed (Example 4). Consider to load the package rrBLUP beforehand as its functions are used over the simulation (Endelman 2011).

**Example 4:** Defining and simulating breeding schemes with “isqg”

> simulation <- function(zzz) {

+ *## (i) inbreeding generations:*

+ F2 <- F1$selfcross(n = N)

+ F2_3 <- unlist(lapply(F2, function(x) x$selfcross(n = P)),

+ *recursive = TRUE)*

+ ## (ii) true breeding values **standardized** and phenotypes:

+ alphas <- scale(sapply(F2_3, function(x) x$alpha(trait)))

+ phens <- rnorm(n = N * P, mean = alphas, sd = sqrt((1 - h2) / h2))

+ *## (iii) VanRaden realized relationship matrix:*

+ G <- A.mat(t(genotype(F2_3)))

+ *## (iv) fitting the mixed models with A and G:*

+ X <- rep(1, N * P)

+ Z <- diag(N * P)

+ mP <- mixed.solve(y = phens, X = X, Z = Z, K = A)

+ mG <- mixed.solve(y = phens, X = X, Z = Z, K = G)

+ *## (v) collecting and delivering statistics:*

+ vgs <- c(vAP = mP$Vu, vAG = mG$Vu) *# additive variance*

+ ves <- c(vEP = mP$Ve, vEG = mG$Ve) *# residuals variance*

+ return(c(VG = vgs, Ve = ves))

+ }

> *## repeating 100 times the simulation*

> output <- sapply(1:100, simulation)

This is a simple but realistic simulation that can be performed with “isqg”, trying to highlight how the simulation can be integrated with third part packages to analyze the simulated data such as rrBlup (Endelman 2011). To reinforce the capabilities of the binary framework, we have found that with a population size of 100 and progeny size of 20 *i.e.*, 2000 simulated individuals, occupy less than one mega byte of memory and 100 replicates of the simulations using only one cpu in a standard laptop spent around 10 min which includes the times to fit the models.

## Discussion

Bitsets are implemented with a limit in size of $\left(2^{32}-1\right)$, which is the upper bound of unsigned long integer numbers in 64 bits architectures. Nevertheless, the variability found within the humans, crop and livestock species *e.g.*, wheat and bovine can be fully represented (Bovine Genome Sequencing and Analysis Consortium 2009; 1000 Genomes Project Consortium *et al.* 2012; International Wheat Genome Sequence Consortium 2018). Taking the largest chromosome of the human genome (248,956,422 BP) with two bits for each base to allow the four possible nucleotides, it is needed 497,912,844 bits which is smaller than the stated limit. In contrast to the representation of biallelic polymorphism by ascii characters (1 byte) or integer numbers (4 bytes) as found in alternative softwares, bits are at least 4 times smaller in modern computing. Moreover, bitwise operations act on chunks of 64 bits that are faster than loops over arrays.

The presented framework was implemented in C++ with an R interface. Access to bitsets is provided by the Boost library, and bit manipulations are carried out by functions in the standard template library. The package is available under a public license (gpl-2). Its low- and high-level interfaces provide great flexibility for users. A sample script showing a basic pipeline with other features is shared as a complementing material simulating a comparison of a few different recombination models.
